# A Novel Localization in Human Large Extracellular Vesicles for the EGF-CFC Founder Member CRIPTO and Its Biological and Therapeutic Implications

**DOI:** 10.3390/cancers14153700

**Published:** 2022-07-29

**Authors:** Francesca Mantile, Matic Kisovec, Giorgia Adamo, Daniele P. Romancino, Matej Hočevar, Darja Božič, Apolonija Bedina Zavec, Marjetka Podobnik, Maria Patrizia Stoppelli, Annamaria Kisslinger, Antonella Bongiovanni, Veronika Kralj-Iglič, Giovanna L. Liguori

**Affiliations:** 1 Institute of Genetics and Biophysics (IGB) “Adriano Buzzati Traverso”, National Research Council (CNR) of Italy, 80131 Naples, Italy; fmantile185@gmail.com (F.M.); mpatrizia.stoppelli@igb.cnr.it (M.P.S.); 2 Department of Molecular Biology and Nanobiotechnology, National Institute of Chemistry, SI-1000 Ljubljana, Slovenia; matic.kisovec@ki.si (M.K.); polona.bedina@ki.si (A.B.Z.); marjetka.podobnik@ki.si (M.P.); 3 Institute for Research and Biomedical Innovation (IRIB), CNR, 90146 Palermo, Italy; giorgia.adamo@irib.cnr.it (G.A.); daniele.romancino@irib.cnr.it (D.P.R.); antonella.bongiovanni@irib.cnr.it (A.B.); 4 Department of Physics and Chemistry of Materials, Institute of Metals and Technology, SI-1000 Ljubljana, Slovenia; matej.hocevar@imt.si; 5 Faculty of Health Sciences, University of Ljubljana, SI-1000 Ljubljana, Slovenia; darja.bozic@biaseparations.com (D.B.); veronika.iglic@fe.uni-lj.si (V.K.-I.); 6 Faculty of Electrical Engineering, University of Ljubljana, SI-1000 Ljubljana, Slovenia; 7 Institute of Experimental Endocrinology and Oncology (IEOS), National Research Council (CNR) of Italy, 80131 Naples, Italy; a.kisslinger@ieos.cnr.it

**Keywords:** extracellular vesicles, microvesicles, CRIPTO, teratocarcinoma, glioblastoma, inhibition of cell migration, cancer therapy

## Abstract

**Simple Summary:**

During tumorigenesis, communication among cells is fundamental. Tumor cells can communicate through the release of extracellular vesicles (EVs), carrying active mediators, that are able to move in the extracellular space and in the body fluids, thereby reaching cells different from the ones of origin. Tumor-derived EVs, upon interaction with tumor target cells, can profoundly change cell characteristics and behavior. Here, we isolated EVs released from human teratocarcinoma cells and we uncovered their ability to inhibit the cell migration of glioblastoma (GBM) cells. Teratocarcinoma EVs contain the oncofetal protein CRIPTO, that is involved in the observed reduction of GBM cell migration. Our results suggest a novel localization and function for CRIPTO in large EVs, and give precious hints for the development of novel therapeutic approaches, based on the control of tumor cell migration, to ultimately improve prognosis and quality of life of GBM patients.

**Abstract:**

Tumor growth and metastasis strongly rely on cell–cell communication. One of the mechanisms by which tumor cells communicate involves the release and uptake of lipid membrane encapsulated particles full of bioactive molecules, called extracellular vesicles (EVs). EV exchange between cancer cells may induce phenotype changes in the recipient cells. Our work investigated the effect of EVs released by teratocarcinoma cells on glioblastoma (GBM) cells. EVs were isolated by differential centrifugation and analyzed through Western blot, nanoparticle tracking analysis, and electron microscopy. The effect of large EVs on GBM cells was tested through cell migration, proliferation, and drug-sensitivity assays, and resulted in a specific impairment in cell migration with no effects on proliferation and drug-sensitivity. Noticeably, we found the presence of the EGF-CFC founder member CRIPTO on both small and large EVs, in the latter case implicated in the EV-mediated negative regulation of GBM cell migration. Our data let us propose a novel route and function for CRIPTO during tumorigenesis, highlighting a complex scenario regulating its effect, and paving the way to novel strategies to control cell migration, to ultimately improve the prognosis and quality of life of GBM patients.

## 1. Introduction

Cancer cell–cell communication is crucial for tumor growth and metastasis. Tumors are very far from being isolated entities, but rather strongly rely on communication between tumoral cells with each other as well as with different types of non-transformed cells which compose the tumor microenvironment. Over the past 10 years, a new paradigm has emerged involving extracellular vesicles (EVs) as key factors for cell communication. EVs are lipid membrane encapsulated structures full of bioactive components released by cells by either blebbing of the cell membrane or by the endocytic pathway. EVs are able to be transported by body fluids and deliver molecules very far from the cell of origin, into target cells in which EVs can induce specific responses [1,2]. Different operational terms are used to distinguish the various subtypes of EVs with respect to physical characteristics (size, density), biochemical composition, or cell of origin. Among the most used terms, there are small EVs (sEVs) and medium/large EVs, m/lEVs, or simply large EVs (lEVs), which indicate vesicles with a size approximately smaller (sEVs) and larger (lEVs) of 200–250 nm (depending also on the type of analysis). lEVs can also reach 1–2 micron in size [2,3]. Due to their intriguing features, EVs emerged as a novel liquid biopsy resource of new biomarkers to be used in cancer diagnosis, prognosis, and treatment as well as promising carriers for the delivery of new therapeutic molecules against cancer.

*CRIPTO* is the founder member of the vertebrate Epidermal Growth Factor-Cripto/FRL-1/Cryptic (EGF-CFC) gene family involved in embryonic and post-natal development [4,5]. In vivo mouse *Cripto* gene targeting revealed a key role of this gene during embryogenesis in A/P polarity definition, mesoderm formation including the mouse primary organizer, early anterior neural patterning, and secondary organizer development [6,7,8,9]. Moreover, the homologous recombination in embryonic stem (ES) cells demonstrated a specific role of Cripto in cardiomyocites differentiation [10,11]. Cripto is also required in extraembryonic tissues for embryo implantation, as demonstrated by the in utero transplantation of embryos with Cripto-depleted trophoblast cells [12]. In the adult, *Cripto* expression levels are largely negligible at the tissue level. The apparent loss of *Cripto* gene expression in adult tissues might reflect the rarity of the cells expressing it, rather than its absence per se [13]. In humans, CRIPTO distribution appears restricted to stem cell niches and the small subpopulation of adult somatic cells, being implicated in stem cell compartment of hierarchically organized tissues (i.e., hematopoietic system, gastrointestinal epithelium), in normal cyclic regenerative processes as well as in injury/regeneration settings [13]. CRIPTO protein is also detectable in the serum of the majority of individuals in a population-based sample [14]. High *CRIPTO* expression levels, instead, have been related to tumorigenesis and poor prognosis in an increasing number of cancers, as for example, melanoma, breast, lung, esophageal, gastric, colon, hepatocellular, pancreatic, renal, prostate, and bladder carcinomas [13,15,16]. In many of these, CRIPTO expression has been mainly detected in cancer stem cells (CSCs), suggesting its role in CSC compartment regulation [13]. On the other side, CRIPTO is also able to induce many tumorigenic features, such as Epithelial-Mesenchymal transition (EMT), tumor cell proliferation, migration, and tumor neovascularization [15,17]. 

Cripto, as with all the EGF-CFC factors, is an extracellular protein characterized by two six-cysteine motifs. The first one is a pseudo EGF-like motif, which differs from the canonical one because of the first two adjacent cysteines and the inability to interact with the EGF receptors; and the second one is a CFC motif, which is unique to this protein family [4,5]. Moreover, Cripto contains at the C-terminus a hydrophobic domain bearing a consensus sequence for a glycosylphosphatidyl inositol (GPI) anchor attachment. GPI anchorage ensures linking to the outer layer of the plasma membrane, and can be also cleaved, releasing extracellularly an active Cripto protein [18,19]. Therefore, Cripto can act both *in cis* as a membrane bound protein as well as *in trans* as a soluble factor. Very recently, Hu and coauthors reported also the presence of CRIPTO on the membrane of the sEVs released by perihilar cholangiocarcinoma (PHCCA) cells [20].

*CRIPTO* was isolated in human teratocarcinoma NTERA2 cells, and, for this reason, it is also named *TERATOCARCINOMA DERIVED GROWTH FACTOR 1* or *TDGF1* [21,22]. Teratocarcinomas are one of the most common types of testicular germ cell tumors in young men, and are composed of embryonal carcinoma stem cells and various layers of differentiated cells [23]. The NTERA2 teratocarcinoma cell line was established from a nude mouse xenograft of the TERA2 cell line, derived from a lung metastasis of a testicular teratocarcinoma. In the presence of retinoic acid (RA), NTERA2 cells differentiate into neuronal postmitotic cells, resembling neural stem/progenitor cell behavior [23]. Therefore, NTERA2 cells have been extensively used as the model system to mimic neural stem/progenitor cells.

GBM is the most common and aggressive primary brain tumor in adults, with a prognosis of 12–15 months and just 3–5% of survival over 5 years [24]. This is mainly because most patients suffer recurrence after treatment that mainly consists of maximal resection followed by radio- and chemotherapy with TMZ [24,25]. Various studies suggested that neural stem cells (NSCs) might be the cells of origin of GBM, which arises from the migration of mutated astrocyte-like NSCs [26,27,28,29]. Previous reports indicated that NSCs target intracranial glioma and could serve as a delivery vector to the anti-glioma therapy [30]. Moreover, a conditioned medium isolated from NSCs, is able to affect GBM cell behavior, by inhibiting glioma cell growth, invasion, and migration in vitro and attenuate the tumor growth in vivo [31]. Interestingly, NTERA2 cells also show a glioma tropism in animal models and were proposed as a cell-based gene delivery, definitely more suitable than NSCs, to inhibit the proliferation and migration of gliomas [32,33]. Moreover, also, a conditioned medium from pristine NTERA2 cells is able to affect GBM development [32].

In the present study, we aimed to investigate the presence of CRIPTO in teratocarcinoma EVs as well as its functional significance. We chose as a model system the NTERA2 teratocarcinoma cell line, as vesicle producing cells, in which *CRIPTO* was originally identified and is strongly expressed, and the U87 GBM cells as target cells to use in functional assays. To our best knowledge, this is the first report on EVs isolated from the NTERA2 teratocarcinoma cells. Functional characterization of the NTERA2 lEVs unraveled a specific role in inhibiting the migration of U87 GBM cells, without affecting cell proliferation or inducing chemioresistance. Our analysis demonstrated the presence of CRIPTO in both NTERA2 sEVs and lEVs, with a strong enrichment in lEVs with respect to the small ones. Finally, our data suggest the involvement of the EV-associated CRIPTO protein in the reduction of U87 GBM cell migration induced by NTERA2 lEVs, paving the way to the possible use of EV-associated CRIPTO as a key player in GBM therapy. 

## 2. Materials and Methods

### 2.1. Cell Culture 

The teratocarcinoma cell line NTERA2 was purchased from ATCC (ATCC-CRL-1973), whereas the glioblastoma cell line U87 was pursed from Merck (89081402, Kenilworth, NJ, USA). Cells were cultured in Dulbecco’s Modified Eagle’s Medium (DMEM) supplemented with 10% (*V*/*V*) fetal bovine serum (FBS), penicillin (100 U/mL), and streptomycin (100 mg/mL) at 37 °C in a humidified atmosphere (5% CO2). All cell culture media and reagents were provided by Gibco (Invitrogen, Carlsbad, CA, USA). For EV isolation, cells were seeded in 150 mm culture plates (Corning, 430599, New York, NY, USA) at a cell density of approximately 1 × 10^4^ cells/cm^2^ (U87) or 2.5 × 10^4^ cells/cm^2^ (NTERA2) and cultured for 24 h as described above. After that, cells were washed twice with phosphate-buffered saline (PBS) and cultured in DMEM supplemented with 10% EV-depleted FBS for 48 h. The EV-depleted FBS was prepared starting from 60% FBS diluted in DMEM by ultracentrifugation at 118,000× *g* for 16 h at 4 °C to remove the EVs present in the serum. After 48 h, cell culture was collected. For each EV preparation, culture medium was collected from about 1.7 × 10^8^ NTERA2 cells or from 7 × 10^7^ U87 cells (used as control for EV isolation). Cell viability at the time of collection ranged from 95% to 100%, estimated by direct cell counting after trypan blue staining. 

### 2.2. Extracellular Vesicle Isolation

EVs were isolated by differential centrifugation [34]. In brief, the conditioned medium was collected in Falcon tubes (Corning Falcon, 352070, New York, NY, USA), and centrifuged twice at 300× *g*, 4 °C for 10 min. Supernatant fractions were further centrifuged twice at 2000× *g*, 4 °C for 10 min to eliminate cell debris. Large extracellular vesicles (lEVs) were pelleted by centrifugation at 10,000× *g* at 4 °C for 30 min in 14 mL polypropylen tubes (Falcon, 352059) in J20 XP Beckman centrifuge, using a J25.50 rotor, followed by washing with PBS. Small extracellular vesicles (sEVs) were pelleted from supernatant by ultracentrifugation at 118,000× *g*, 4 °C for 70 min, in 38.5 mL polypropylen tubes (Beckmann coulter, 326823, Brea, CA, USA) in Optima XE, Beckman ultracentrifuge using SW28 rotor, followed by washing with PBS. lEVs and sEVs were resuspended in 50 μL PBS for further analysis. The protein content of EV samples was measured using the BCA Protein Assay Kit (Thermo Fisher Scientific, Waltham, MA, USA).

### 2.3. Cell Lysate Preparation 

Cells were lysed with a Radioimmunoprecipitation Assay (RIPA) buffer (50 mM Tris·HCl at pH 8, 150 mM NaCl, 1 mM EDTA pH 8, 1% Triton X-100) supplemented with Complete Protease Inhibitor Mixture tablets (Roche Diagnostics S-4693159001, Basilea, Switzerland). The protein concentration of cell lysates was measured using the Bradford Protein Assay (Biorad, Segrate, Italy).

### 2.4. Western Blot

Samples in reducing the Laemmli buffer were boiled at 95–100 °C for 5 min and separated by sodium dodecyl-sulfate polyacrylamide gel electrophoresis (SDS-PAGE) (12%). Then, proteins were transferred to polivinilidenfluoro (PVDF) membranes, and the membranes were blocked with 5% milk in TBS-T (50 mM Tris HCl pH 8.0, 150 mM NaCl with 0.05% Tween 20) for 1 h at room temperature, followed by incubation overnight at 4 °C with the following primary antibodies: anti-CRIPTO rabbit monoclonal (1:500 dilution, Abcam, 133236, Cambridge, UK); anti-HSP70 mouse monoclonal (1:500 dilution, Santa Cruz Biotechnology, clone W27, Dallas, TX, USA); anti-βActin rabbit polyclonal (1:1000 dilution, SIGMA, A2066, Saint Louis, MO, USA); Calnexin rabbit polyclonal antibody (1:500 dilution, NovusBio NB100-1965); CD63 rabbit polyclonal antibody (1:500 dilution, Invitrogen, #PA5-92370, Waltham, MA, USA) in 5% milk in TBS-T. After washing in the TBS-T buffer, the membranes were incubated for 1 h at room temperature with a 1:10,000 dilution of goat anti-rabbit secondary antibody (SIGMA, 12-348) or anti-mouse secondary antibody in 5% milk in TBS-T. Membranes were then washed three times in the TBS-T buffer and chemiluminescence detection was performed using an enhanced Chemiluminescence Kit according to the manufacturer’s protocol (Clarity Western ECL substrate, Biorad, 1705060, Segrate, Italy).

### 2.5. Scanning Electron Microscopy

Isolates were fixed overnight at 4 °C in Karnovsky’s fixative with modification composed of 2.5% glutaraldehyde, 0.4% formaldehyde, and phosphate buffer saline (PBS) at pH 7.4 (137 mM NaCl, 2.68 mM KCl, 10.14 mM Na_2_HPO_4_, and 1.84 mM KH_2_PO_4_), and post-fixed the following day with OsO_4_, according to the protocol adopted from Lešer et al. 2007 [35]. Fixatives were removed in three rinsing steps using PBS (10 min incubation in each step). Then, samples were incubated in 2% OsO_4_ for one hour, rinsed three times with distilled water (10 min incubation time in each step), and with saturated water solution of thiocarbohydrazide (15 min incubation time), rinsed three times with distilled water (10 min incubation time in each step), incubated again in 2% OsO_4_ for 1 h, washed three times with distilled water (10 min incubation time in each step), and dehydrated in graded series of ethanol (30–100%, 10 min in each solution). Absolute ethanol was replaced three times, treated by the graded series of hexamethyldisilazane (mixed 30% and 50% with absolute ethanol and pure, 10 min incubation time in each step), and finally air dried overnight. Fixed and dehydrated samples were Au/Pd coated by Precision Etching and Coating System or PECS (Gatan Inc 682, Pleasanton, CA, USA), and examined using a JSM-6500F Field Emission Scanning Electron Microscope (JEOL Ltd., Tokyo, Japan).

### 2.6. Nanoparticle Tracking Analysis

Nanoparticle size distribution and concentration were measured using a NanoSight NS300 (Malvern Panalytical, Malvern, UK). The instrument was equipped with a 488 nm laser, a high-sensitivity sCMOS camera, and a syringe pump. The lEVs and sEVs were diluted to generate a dilution in which 20–120 particles per frame were tracked to obtain a concentration within the recommended measurement range (1–10 × 10^8^ particles/mL). The results were based on five and two independent samples for NTERA2 and U87, respectively. For each sample, 5 experiment videos of 60 s duration were analyzed using a nanoparticle tracking analysis NTA 3.4 Build 3.4.003 (camera level 15–16) with syringe pump speed 60. A total of 1500 frames were examined per sample, which were captured and analyzed by applying instrument-optimized settings using a suitable detection threshold so that the observed particles were marked with a red cross and that no more than five blue crosses were seen. Further settings such as blur size and Max Jump Distance were set to “automatic” and viscosity was set to water (0.841–0.844 cP).

### 2.7. Cryogenic Transmission Electron Microscopy 

Quantifoil^®^ R 2/2 (or 1.2/1.3), 200 (Quantifoil Micro Tools GmbH, Großlöbichau, Germany) EM grids were glow discharged for 60 s at 20 mA and positive polarity in air atmosphere (GloQube^®^ Plus, Quorum, Laughton, UK). Vitrobot conditions were set to 4 °C, 95% relative humidity, blot time: 5 s, and blot force: 4. An amount of 2 µL of the sample suspension was applied to the grid, blotted, and plunge-frozen in liquid ethane with Vitrobot Mark IV (Thermo Fisher Scientific, Waltham, MA, USA). Samples were visualized under cryo conditions with a Falcon 3EC detector on a 200 kV microscope Glacios (Thermo Fisher Scientific, Waltham, MA, USA).

### 2.8. Flow Cytometry

NTERA2 cells were detached using 0.5% trypsin, counted, and resuspended in 5% FBS in PBS (1 × 10^6^ cells were resuspended in a volume of 100 ul) and incubated with the primary anti-CRIPTO rabbit polyclonal antibody (Abcam, 19917) for 1 h in ice at dark. After washing twice with PBS, cells were incubated with the secondary antibody Alexa Fluor 594 donkey anti-rabbit (Invitrogen A21207) for 1 h in ice at dark. After washing twice with PBS, cells were resuspended in 5% FBS in PBS, filtered, and analyzed with the BD FACS ARIAIII flow cytometer. Data were collected using BD FACSDiva software (v8.0.1 Becton Dickinson, Franklin Lakes, NJ, USA). For each sample, the data of 20,000 cells were collected.

### 2.9. Wound Healing Assay

U87 cells were seeded into Ibidi inserts (Ibidi, Gmbh, Martinsried, Germany, 81176), and placed in 24-well plates (13,000 cell/well) in the complete medium (10% FBS; 100 U/mL penicillin and 100 mg/mL streptomycin in DMEM). After 24 h, the Ibidi inserts were removed, the cells were washed with PBS, and the complete medium was replaced with DMEM 2% EV-depleted FBS, penicillin (100 U/mL), and streptomycin (100 mg/mL). The following treatments were added to cells: 10 μg/mL of NTERA2-lEVs; 10 μg/mL of NTERA2- lEVs previously incubated 1 h at 4 °C with 1:100 anti-CRIPTO antibody (Abcam, ab 19917) (lEVs-α-CRIPTO); 10 μg/mL of NTERA2- lEVs previously incubated 1 h at 4 °C with 1:100 anti-rabbit IgG antibody (SIGMA, 12-348) (lEVs-α-IgG). As the control, non-treated cells were used. The closing of the wound was observed after 6 and 20 h. All treatments were performed in the presence of 2 ug/mL mitomycin C to inhibit cell proliferation. ImageJ software (v1.51, Wayne Rasband, National Institutes of Health, USA, open source) was used to analyze the results. The relative wound healing area was calculated as (initial area—final area)/initial area. Three independent experiments were performed, each one in duplicate. Data are shown as means ± standard error of the mean (s.e.m).

### 2.10. Cell Proliferation and Drug Sensitivity Assay

U87 cells were seeded in a volume of 100 μL at 5 × 10^2^ cells/well in 96-well plates. After 24 h, the culture medium was replaced with DMEM 2% EV-depleted FBS, penicillin (100 U/mL), and streptomycin (100 mg/mL), and the following treatments were added to cells: 10 μg/mL of NTERA2-lEVs; 10 μg/mL of NTERA2-lEVs previously incubated 1 h at 4 °C with 1:100 anti-CRIPTO antibody (Abcam, 19997) (lEVs-α-CRIPTO); 10 μg/mL of NTERA2- lEVs previously incubated 1 h at 4 °C with 1:100 anti-rabbit IgG antibody (SIGMA, 12-348) (lEVs-α-IgG). After incubation at 37 °C for 48 and 72 h, 20 μL of CellTiter 96^®^ AQueous One Solution Reagent (Promega) was added, and the plates were incubated at 37 °C for 2 h. The reaction was stopped by the addition of 25 μL 10% SDS and the absorbance (A) at 490 nm was measured using a microtiter plate spectrophotometer. Values were expressed as means ± s.e.m. of two independent experiments, each one performed in duplicate. 

In drug sensitivity experiments, freshly prepared 100 μM of Temozolomide (TMZ; Sigma) was added to cells, in combination or not with NTERA2- lEVs. After incubation at 37 °C for 72 h, 20 μL of CellTiter 96^®^ AQueous One Solution Reagent (Promega) was added, and the plates were incubated at 37 °C for 2 h. The number of viable cells in each sample was calculated as the percentage of viable cells after the treatment, with respect to the one found in the control with no TMZ. 

### 2.11. Quality Management 

The study has been performed in compliance with the VES4US Quality Management System [36] and following minimal instruction for studying the EVs (MISEV2018) [3]. Cell culture experiments have been conducted in accordance with the quality-based research guidelines previously identified [37,38].

### 2.12. Statistical Analysis

Student’s *t*-test was used to determine the statistical significance of the quantitative results. Statistical significance was indicated by * = *p* < 0.05; ** = *p* < 0.01; *** = *p* < 0.001.

## 3. Results

### 3.1. Isolation of Extracellular Vesicles from NTERA2 Teratocarcinoma Cells 

**Figure 1:**
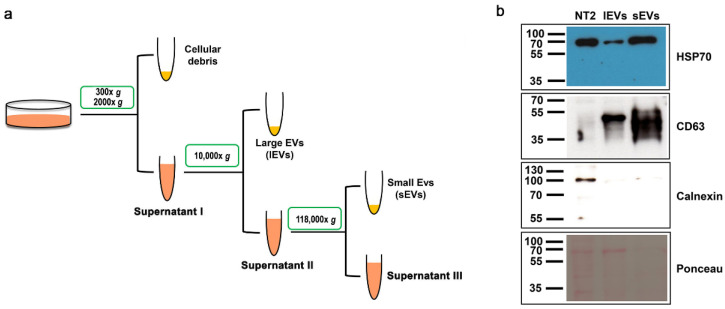
Isolation of extracellular vesicles from NTERA2 cells. (**a**) General outline of the differential centrifugation-based protocol used. (**b**) Protein immunoblotting with the antibody against the EV specific markers heat shock protein 70 (HSP70) and CD63, and the negative EV marker calnexin. Equal protein amounts (20 ug) of NTERA2 cell lysates (NT2), large extracellular vesicle (lEV), and small extracellular vesicle (sEV) preparations were analyzed. On the bottom: the filter after protein transfer and Red Ponceau protein revelation.

To investigate EV production by the human pluripotent teratocarcinoma NTERA2 cell line, we applied a differential centrifugation method to isolate two different fractions of EVs from NTERA2 conditioned culture medium, conventionally corresponding to sEVs and lEVs (Figure 1a). We analyzed by Western blot the EV preparations obtained, using antibodies to EV specific markers, both cytosolic and transmembrane, as well as the negative EV marker calnexin (Figure 1b), as recommended by MISEV 2018 [3]. Both large and small EV preparations were positive for the EV associated markers, the cytosolic heat shock protein 70 (HSP70), and the transmembrane CD63 tetraspanin, and negative for the presence of the endoplasmic reticulum-associated protein calnexin (Figure 1b).

**Figure 2:**
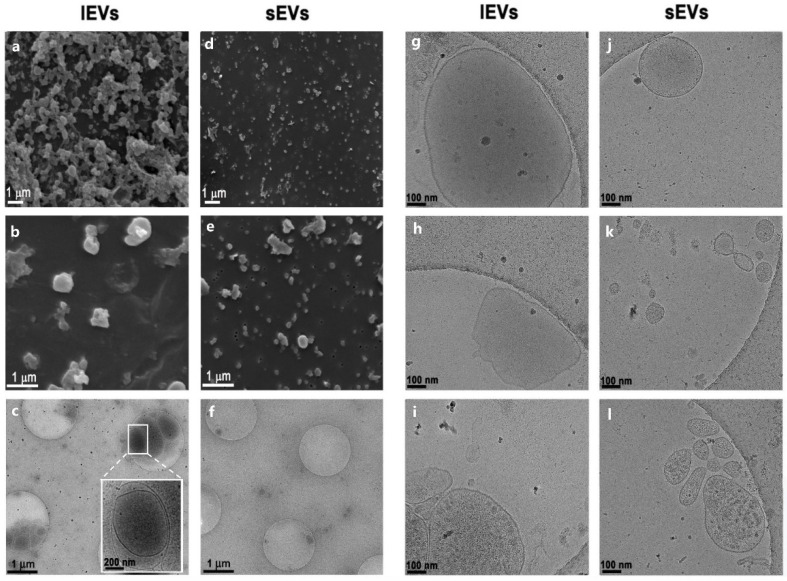
Analysis of NTERA2 cell-derived extracellular vesicles by electron microscopy. Scanning electron microscopy (**a**,**b**,**d**,**e**) and cryogenic transmission electron microscopy (**c**,**f**,**g**–**l**) images of large and small extracellular vesicle (lEV and sEV) preparations. Magnification bars are reported in each image.

We further analyzed NTERA2 EV preparations by scanning electron microscopy (SEM) and cryogenic transmission electron microscopy (Cryo-TEM), as reported in Figure 2. The SEM analysis highlighted the presence of particles in both lEV and sEV samples, differing in both size and abundance (Figure 2a,b,d,e). Cryo-TEM images evidenced the presence of particles delimited by a lipidic bilayer (Figure 2g–l), thereby proving that these particles were membrane-enclosed vesicles. The different Cryo-TEM images shown in the panel confirmed the difference in size between lEVs and sEVs and showed also the heterogeneity, in terms of size and shape, inside each EV type of samples (Figure 2j–l). Interestingly, the content of lEVs was mostly opaque, suggesting the presence of electron dense material inside or on the surface of the vesicles. In particular, some lEVs also harbored inside smaller vesicles (Figure 2c).

**Figure 3:**
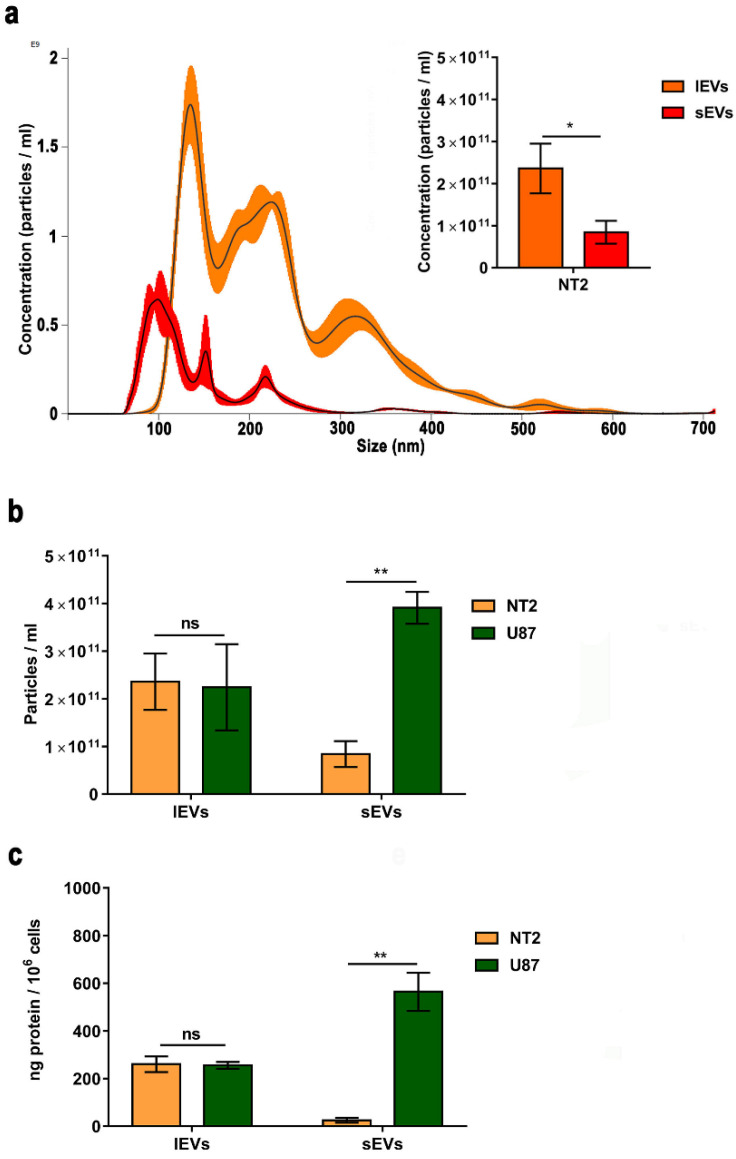
Extracellular vesicle quantification. (**a**) Nanoparticle tracking analysis (NTA) of NTERA2 extracellular vesicles. Representative image of size distribution of small extracellular vesicles or sEVs (red line) and large extracellular vesicles or lEVs (orange line). The inset histogram shows the mean ± standard mean error of sEV and lEV concentration. (**b**,**c**) Comparison of the concentration of particles measured by NTA (**b**) and the protein yield (**c**) in sEV and lEV samples isolated from NTERA2 and U87 cancer cells. The volume of resuspension is kept constant. To determine particle concentration, five and two different samples have been analyzed for NTERA2 and U87, respectively. For each sample, five experiment videos of 60 s duration were analyzed. To determine protein yield six and three different experiments have been analyzed for NTERA2 and U87, respectively. ns not significant, * *p* < 0.05; ** *p*< 0.01.

We also characterized the NTERA2 EV preparations by NTA (Figure 3a). The NTA-profiles of the size distributions of small and large NTERA2-derived EVs indicated a canonic distribution for the sEVs, in which the main population was about 100 nm (mean at 151 ± 3 nm), while the large EVs had a distribution in a wider size in which two other distinct populations were visible, one about 250 nm and another about 320 nm (mean at 230 ± −3 nm). From the inset histograms in Figure 3a, which show a representative result of five NTA measurements on five independent NTERA2-derived vesicle preparations, it is evident that lEVs (average concentration equal to 2.36 × 10^11^ ± 2.95 × 10^10^ particles/mL) were 2.81 times more concentrated than the sEVs (average concentration equal to 8.42 × 10^10^ ± 3.01 × 10^9^ particles/mL). All together, these data demonstrated that the preparations isolated from NTERA2 cells through the widely used differential centrifugation method truly contained vesicles. Both sEVs and lEVs were heterogeneous in size, shape, and density. Moreover, the amount of NTERA2 lEVs isolated was almost three time higher than the sEV amount. 

As a comparison, we also applied the same methodology to isolate sEVs and lEVs from the U87 GBM cell line, whose EVs have been well characterized in the literature [39]. As shown in Figure 3b, the concentration of particles collected from NTERA2 and U87 cancer cell lines was very similar in the case of lEVs (2.36 × 10^11^ ± 2.95 × 10^10^ particles/mL and 2.24 × 10^11^ ± 9.06 × 10^10^, respectively). Interestingly, the concentration of particles inside the NTERA2 sEV samples (8.42 × 10^10^ ± 3.01 × 10^9^ particles/mL) was significantly lower with respect to those isolated from U87 cells (3.90 × 10^11^ ± 6.36 × 10^10^ particles/mL). By comparing the protein yield of the EV preparations from both cancer cell cultures, we found a similar trend (Figure 3c). The protein yield of NTERA2 and U87 lEV fractions was very similar (260.4 ± 33 ng/10^6^ cells and 255.9 ± 14.7 ng/10^6^ cells, respectively), whereas the protein yield associated with NTERA2-derived sEVs (25.2 ± 25.3 ng/10^6^ cells) was very much lower with respect to U87-derived sEVs (564.6 ± 79.9 ng/10^6^ cells). 

### 3.2. Functional Characterization of NTERA2 Large Extracellular Vesicles 

Being that the lEVs isolated from NTERA2 were more abundant than the correspondent sEVs, we preferred to focus on lEV functional characterization. Therefore, we tested their effect on different tumor features, such as tumor cell migration, proliferation, and drug-sensitivity, using as a model system the well-characterized U87 human GBM cell line [40].

**Figure 4:**
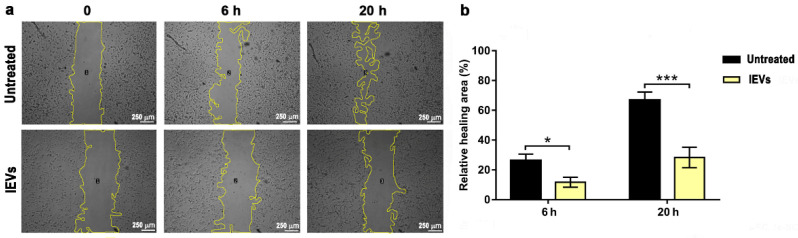
Wound healing assay. (**a**) Images of U87 cells, both untreated and treated with NTERA2 large extracellular vesicles (lEVs) were taken at 0, 6, and 20 h after wound. The open area surrounded by a yellow line is the one calculated by the ImageJ software. (**b**) Relative wound healing area in the different conditions. Three independent experiments were performed, each one in duplicate. Data are shown as means ± standard error of mean. * *p* < 0.05; *** *p* < 0.001.

First, we tested the effect of lEVs on tumor cell migration, by using the wound healing assay (Figure 4a), as described in Materials and Methods. In the absence of lEVs, U87 wound closure was almost completed after 20 h. Noteworthy, the incubation of U87 GBM cells with NTERA2 lEVs affected U87 tumor cell migration. U87 cell cultures showed a delay in wound closure in the presence of lEVs, which was detectable after 6 h and even more striking after 20 h of treatment (Figure 4b). These results uncovered the ability of NTERA2 cell-derived lEVs in impairing GBM cell migration.

**Figure 5:**
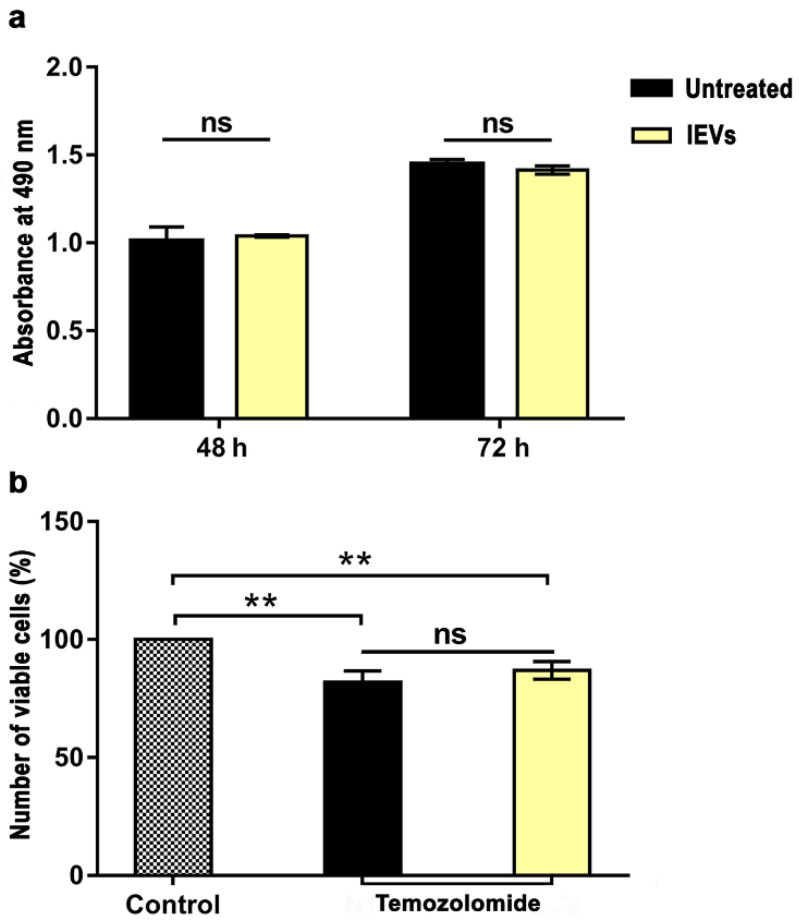
Cell proliferation and drug-sensitivity assay. (**a**) Cell proliferation assay. Absorbance of colorimetric assay of U87 cells after 48 and 72 h in absence (untreated) or presence of NTERA2 large extracellular vesicles (lEVs). No significant (ns) difference between the two samples was detected. (**b**) Temozolomide (TMZ) drug-sensitivity assay after 72 h of treatment, in combination or not with NTERA2 lEVs. Cell viability decreased with TMZ treatment, but no significant differences were detected between the effect caused by TMZ alone or in combination with NTERA2 lEVs. The results are shown as means ± standard error of mean from two independent experiments, each one in duplicate. ns not significant, ** *p* < 0.01.

Then, we analyzed the effect of lEVs on GBM cell proliferation and drug sensitivity by measuring the number of viable cells in the different conditions through the CellTiter 96^®^ AQueous One Solution Reagent colorimetric assay. Incubation of U87 cells with the NTERA2 lEVs for 48 and 72 h did not cause any significant variation in the absorbance measured, and then in the number of viable cells, with respect to U87 untreated cells (Figure 5a). These data indicate that lEVs did not significantly alter the U87 GBM cell proliferation rate. 

In the drug sensitivity assay, lEV incubation was coupled with treatment with the chemotherapeutic agent TMZ, mostly employed in GBM therapy (Figure 5b). As expected, TMZ drug treatment reduced U87 cell viability with respect to the control, but lEVs did not cause any significant change in the sensitivity of U87 GBM cells to the chemotherapeutic agent. 

All together, these data point to a specific inhibitory effect of NTERA2-derived lEVs on U87 tumor cell migration, without affecting cell proliferation and sensitivity to the TMZ drug.

### 3.3. Association and Functional Relevance of CRIPTO in NTERA2 Extracellular Vesicles 

**Figure 6:**
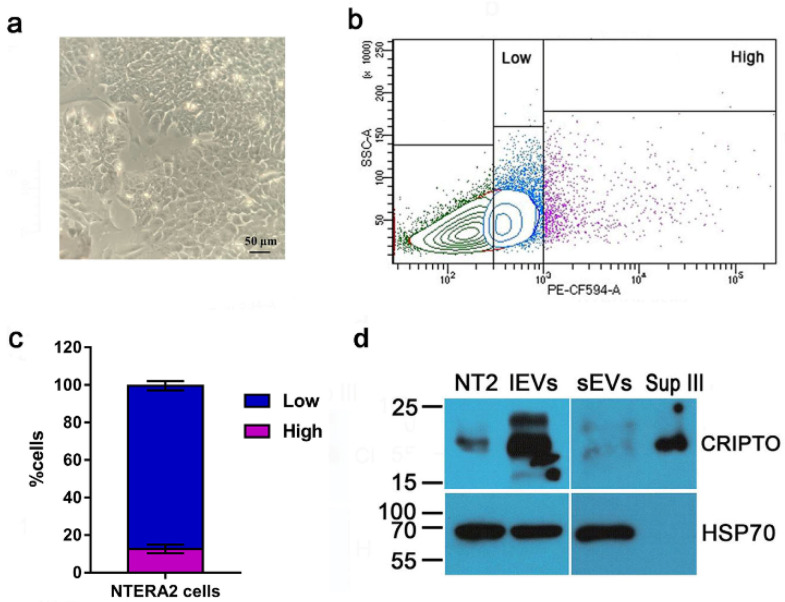
CRIPTO protein distribution in NTERA2 cell line. (**a**) Representative image of NTERA2 cell culture. (**b**) Flow cytometry analysis of CRIPTO protein presence on the cell membrane of NTERA2 cells. Two populations of CRIPTO positive cells (high and low), exposing different levels of the protein on the membrane, were distinguished by the respective gates. (**c**) Distribution of the CRIPTO high and low populations. The results are a mean of three independent experiments. (**d**) Equal protein amounts (20 ug) of NTERA2 cell lysates (NT2), large extracellular vesicles (lEVs), small extracellular vesicles (sEVs), and supernatant at the end of the centrifugation procedure (Sup III) were immunoblotted with an antibody against CRIPTO or against the EV marker heat shock protein 70 (HSP70).

NTERA2 cells are characterized by a strong expression of the oncofetal *CRIPTO* gene, that has been isolated precisely in these cells, and for this reason was also named *TERATOCARCINOMA DERIVED GROWTH FACTOR 1* or *TDGF1* [21,22]. First of all, we verified that in our culture conditions NTERA2 cells expressed *CRIPTO* and exposed the CRIPTO protein on the plasma membrane. A flow cytometry analysis (Figure 6b,c) highlighted the heterogeneity of the NTERA2 cell culture, in which it was possible to detect cells exposing on the plasma membrane different amounts of CRIPTO protein. By defining the gates shown in Figure 6b, it was possible to distinguish a high- and low-expressing population, corresponding approximately to the 12.55 ± 2.35 % and 86.95 ± 2.45 %) of NTERA2 CRIPTO positive cells, respectively. These results might suggest a dynamic equilibrium, possibly due to modifications involving the plasma membrane, such as, for example, the known mechanism of CRIPTO GPI-anchor cleavage and extracellular release or an unprecedented CRIPTO-linked mechanism of vesicle budding from the cell membrane. 

Therefore, we analyzed by Western blot whether CRIPTO was associated to the EVs released by NTERA2 cells (Figure 6d). As expected, CRIPTO positive signals were detected in the NTERA2 cell lysate as well as in the non-particulate supernatant (Sup III) following EV separation. This finding was expected in view of the GPI-anchored nature of CRIPTO, that can be either membrane-associated or released as a soluble protein in the extracellular milieu. Noteworthy, CRIPTO was also detected in the EV preparations, both lEV and sEV, and, in particular, was strongly enriched in the lEVs (Figure 6d). 

**Figure 7:**
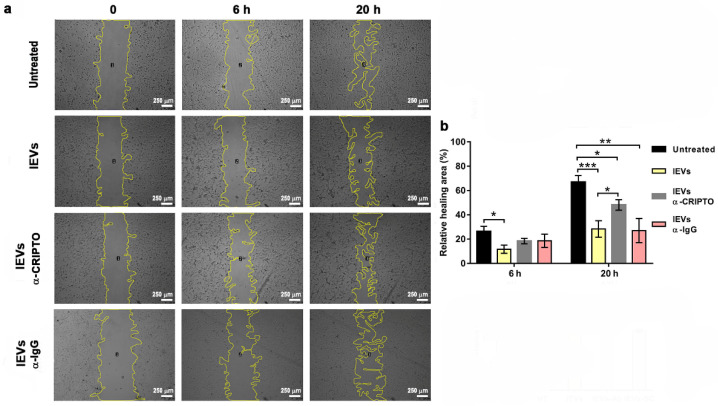
Rescue of U87 cell migration. (**a**) Cell migration at 0, 6, and 20 h of untreated U87 cells, U87 treated with NTERA2 large extracellular vesicles (lEVs) or NTERA2-lEVs previously incubated with anti-CRIPTO antibody (lEVs-α-Cripto) and a scramble antibody (lEVs-α-IgG), respectively. Open area is outlined by a yellow line and analyzed with ImageJ. (**b**) Relative wound healing area in the different conditions. Three independent experiments were performed, each in duplicate. Data are shown as means ± standard error of mean. Inhibitory effect of NTERA2-lEVs was partially rescued after 20 h when vesicles were pretreated with anti-CRIPTO antibody. Rescue was not observed when anti-IgG antibody was added. * *p* < 0.05; ** *p*< 0.01; *** *p* < 0.001.

The enrichment of CRIPTO in lEVs led us to hypothesize that the CRIPTO protein might be involved in the reduction of U87 cell migration induced by NTERA2 lEVs (Figure 4). To test this hypothesis, we repeated the wound healing experiments by preincubating the lEVs with an antibody specific for CRIPTO that binds to the native CRIPTO protein. We found that preincubation with a CRIPTO antibody decreased the effect of lEVs and partially rescued cell migration (Figure 7). Preincubation with a nonspecific antibody, instead, did not cause any effect (Figure 7). We tested the effect of the preincubation of lEVs with a CRIPTO antibody also in cell proliferation and TMZ drug-sensitivity assays, and we could not find any difference with respect to pristine lEVs.

## 4. Discussion

Cancer cells can communicate with each other and induce phenotype changes in the recipient cells. One of the mechanisms by which they can communicate involves cancer EV release and uptake. The EV exchange between cancer cells with different phenotypic properties has been shown to transfer apoptosis resistance [41], drug resistance [42], as well as metastatic and migration properties [43]. EVs can be released by the direct outward budding and fission of the plasma membrane, and in this case are generally called microvesicles or ectosomes, or have an endocytic origin, being formed as intraluminal vesicles (ILVs) of the late endosomes or multivesicular bodies (MVBs), and then are named exosomes [2,44]. The diameter of exosomes is close to the diameter of ILVs from which they derive, therefore ranging from 30 to 100–150 nm in diameter, whereas microvesicle budding from plasmatic membrane escapes from this size restriction. Microvesicles, then, are more heterogeneous and they can be as small as exosomes or reach 1–2 μm in diameter [2,44,45,46]. During interaction with target cells, components of the EV membranes can bind directly to receptors [2,47,48]. Alternatively, EV cargo is internalized into the cell through the direct fusion of the EV with the plasma membrane and the release of the EV content in the cytoplasm, or through endocytosis/phagocytosis pathways in which intact EVs fuse into the MVBs [44,49,50,51].

Even though NTERA2 teratocarcinoma cells have been extensively characterized, we found no study in the literature on EVs released by these cells or other teratocarcinoma cell lines. Our work isolated and characterized for the first time the EVs released by NTERA2 cells, showing a significant lower number of particles and protein yield in the sEV preparations obtained compared to the lEV ones. These data suggest that NTERA2 cells might preferentially release larger vesicles, and then led us to speculate that the preferential NTERA2 EV biogenesis mechanism might involve outer membrane budding of microvesicles, with respect to the endocytic pathway involved in generating smaller vesicles. Therefore, NTERA2 cells might be an interesting model system for the study of microvesicle biogenesis and their specific components. A cryogenic TEM revealed also the presence of multilayered EVs which have previously been described in the literature as isolated from different sources such as blood plasma [52], semen [53], conditioned media of cultured cells [54], and also in suspensions of lipid vesicles [55]. Different mechanisms leading to the multilayered vesicles were suggested, including the nuclear or mitochondrial origin of EVs and membrane fusion or fission [53]. It should, however, be considered that multilayered EVs could be formed also due to the loss of water during sample preparation [55]. In fact, as the volume of the vesicle decreases while its surface remains constant, its relative volume decreases, and invaginations might form and develop into concentric vesicles [56].

Interestingly, our data point to a functional role of the lEVs released by NTERA2 cells on GBM development, in particular by counteracting GBM cell migration, without a notable effect on cell proliferation and chemoresistance. The effect of NTERA2 lEVs might be further investigated by using different migration assays (i.e., transwell assay) as well as in animal models to analyze their potential as migratory inhibitors of GBM cells in vivo.

Moreover, our findings show, for the first time, the association of the CRIPTO protein to lEVs. The ability of the antibody specific for CRIPTO to rescue the antimigratory effects induced by NTERA2 lEVs has two important implications. The first one is that CRIPTO is accessible to the antibody and then exposed on the surface of lEVs. CRIPTO is a GPI-anchored protein linked to the outer layer of the cell membrane [18,57] and localized, as with most GPI proteins, to the lipid rafts domains, characterized by a peculiar lipidic composition and involved in membrane blebbing and microvesicle release [58,59]. Therefore, the membrane GPI-anchored CRIPTO protein can be present on the surface of NTERA2 microvesicles which bud from the plasma membrane. We also found CRIPTO associated to sEV fractions, though at a lesser extent. In agreement, several studies have reported the presence of various GPI-anchored proteins also in exosomes [60]. In particular, very recently CRIPTO has been found on the surface on sEVs released by another type of cancer cell: the perihilar cholangiocarcinoma (PHCCA) cells [20]. Our analysis showing CRIPTO also in sEVs and lEVs derived from teratocarcinoma cells, coupled with the previous finding of Hu and coauthors (2021) [20], led us to hypothesize a more general mechanism that could involve many types of cancer. Noteworthy, CRIPTO might be an EV marker present on the surface of vesicles released by different types of cancer cells. 

The second implication is that the lEV-associated form of CRIPTO plays a key role in the negative effect on GBM cell migration induced by NTERA2 lEVs. These findings are really intriguing, in consideration that previous studies demonstrated the ability of a functionally active recombinant soluble CRIPTO protein to stimulate the migration of different types of cells, including mammary epithelial cells and human umbilical vein endothelial cells [61,62,63]. The induction of CRIPTO shedding from the cell membrane by the Glycosylphosphatidylinositol-Phospholipase enzyme is also able to enhance endothelial cell migration [63]. More strikingly, most literature on CRIPTO pointed to its role as an oncofetal protein associated with increased cancer features and the worst patient prognosis [15,16,64]. However, we previously unmasked a dual role of Cripto in tumorigenesis, by showing that *Cripto* haploinsufficiency in mouse models, surprisingly, increased colon tumor formation after azoxymethane treatment [65]. These data suggested a role of Cripto in tumorigenesis that is intricately regulated and strictly dependent on the cellular context in which it acts, as well as the balance with other molecules in the same signaling pathway, such as the Glucose Regulated Protein 78 kDa (GRP78) [65]. 

As regards GBM, despite the first evidence of a role of CRIPTO in this type of cancer [66,67], its significance in GBM progression and pathogenesis has not been addressed so far. Recent studies focused on U87 GBM cells, in which, however, *CRIPTO* was overexpressed to study its functional effect, reporting enhanced U87 cell proliferation and migration after *CRIPTO* overexpression [68,69]. A role of CRIPTO in U87 cellular migration was also suggested by localization studies, in which, after its overexpression, the CRIPTO protein was mainly sub-localized in tunneling nanotubes, dynamic filopodia, and shed filopodia/retraction fibers [70], which are dynamic structures known to be involved in cell orientation and migration [71]. The overexpression studies definitely validated the use of the U87 as an ideal cellular system in which to test exogenous CRIPTO activity. In our work, however, we adopted a more physiological approach based on U87 cell exposure to CRIPTO through incubation with natural EVs intrinsically strongly enriched in CRIPTO. Our findings highlight a specific role of lEV-associated CRIPTO in GBM cell migration. However, differently from previous studies, the CRIPTO-enriched lEV treatment causes a reduction in GBM cell migration. Our data add a higher grade of complexity for the fine tuning of CRIPTO localization and consequent cellular function. CRIPTO is able to interact with a plethora of molecules on the cell membrane, activating different signaling pathways and cellular responses [17,64]. The U87 cells contain most of the surface molecules to whom CRIPTO binds; in fact, they expose on the membrane the main CRIPTO interactors that are ALK4 and ALK7 receptors, being able to activate in the U87 the downstream Smad signaling pathway [72], Glypican 1 [73], as well as the Wnt co-receptors low-density lipoprotein receptor-related protein LRPs [74], Notch1 [75], and GRP78 [76]. It would be tempting to speculate that the lEV-associated CRIPTO protein might act as a dominant negative able to bind and sequester in an inactive form the target receptor complexes on the U87 cell surface, thus altering the downstream cellular response. In a more general scenario, EV-CRIPTO might interfere with the activity of the other CRIPTO forms (the plasma membrane bound and the soluble ones), and the balance among the different CRIPTO forms might account for the resultant effect of CRIPTO as a tumor agonist or antagonist.

Interestingly, other signaling molecules that interact with CRIPTO, such as the soluble factors Transforming Growth Factor β (TGFβ) or Lefty, induce a pleiotropic reaction that leads to a diverse and varied set of responses that range from cytostatic and apoptotic tumor-suppressive ones, to proliferative, invasive, angiogenic, and oncogenic ones [77,78,79,80]. The pleiotropic pathway is modulated by the cellular context and its integration with other signaling pathways [79]. In addition, TGFβ itself, other ligands of the TGFβ superfamily, as well as key components of the TGFβ signaling machinery, including GRP78, have been found associated to EVs [81,82]. The use of alternative routes might serve the purpose of finely regulating the role of these factors in cancer development and progression. Finally, their association to EVs might also be exploited for cancer diagnostic and therapeutic purposes. 

All together, our results expand the knowledge of CRIPTO localization and function, paving the way to the possible exploitation of NTERA2 CRIPTO positive EVs for GBM therapeutic approaches aimed to reduce GBM cell migration. Since migration and infiltration in healthy brain tissues constitute one of the main causes of the bad GBM prognosis, novel strategies aimed to reduce GBM cell migration might potentially have a strong impact on GBM treatment.

## 5. Conclusions

In this study, we isolated and characterized for the first time EVs released by the human pluripotent teratocarcinoma NTERA2 cells, uncovering the association of CRIPTO to lEVs. Furthermore, NTERA2-produced lEVs, when incubated with U87 GBM cells, are able to reduce their migration, in a CRIPTO-dependent manner, without inducing cell proliferation and chemioresistance. These results pave the way for further investigations on the development of novel therapeutic approaches to reduce GBM cell migration and enhance the effectiveness of GBM treatments, thus improving prognosis and quality of life of GBM patients. 

## 6. Patents

Italian patent number I0197620 has been filed.

## Data Availability

Data are contained within the article. Additional data are available from the corresponding author upon reasonable request.
